# Safety, tolerability, and pharmacokinetics of a single ascending subcutaneous dose of GSK3772847 in healthy participants

**DOI:** 10.1002/prp2.1054

**Published:** 2023-02-27

**Authors:** Eleni Pefani, Sally Stone, Chang‐Qing Zhu, Carol Nunn, David Fairman

**Affiliations:** ^1^ GSK Medicines Research Centre Stevenage Hertfordshire UK; ^2^ GSK Brentford Middlesex UK; ^3^ GSK RD Respiratory R&D Brentford Middlesex UK

**Keywords:** asthma, monoclonal antibodies, pharmacodynamics, pharmacokinetics, Phase 1

## Abstract

The aim of this study was to evaluate the safety, tolerability, pharmacokinetics (PK), and pharmacodynamics (PD) of GSK3772847, compared with placebo administered subcutaneously (SC) in healthy participants, including cohorts of Japanese and Chinese participants. This was a single‐center, randomized, placebo‐controlled, double‐blind, single ascending dose study. Following a screening period of up to 28 days, eligible participants were assigned to one of four cohorts receiving a single dose of GSK3772847 70 mg (cohort 1) or 140 mg (cohorts 2, 3, and 4) or placebo SC. In cohorts 1 and 2, participants were randomly assigned to one of three injection sites (upper arm, abdomen, or thigh), while cohorts 3 and 4 included Japanese and Chinese participants, respectively, assigned to receive GSK3772847 or placebo SC (upper arm). Participants attended follow‐up visits on Days 9, 15, 29, 43, 57, 71, and 85 before final analysis. GSK3772847 was generally well tolerated. Most adverse events (AEs) were mild, resolved without treatment and were considered not related to study treatment by the investigator. There were no serious AEs or deaths during the study. The PK and PD were dose dependent, with negligible differences across injection sites or ethnicities. Target engagement was demonstrated by reduced free soluble interleukin 33 (sIL‐33) concentrations and substantially increased total sIL‐33 concentrations compared with baseline. Subcutaneously administered GSK3772847 was well tolerated in healthy participants, including cohorts of Japanese and Chinese participants, and shows consistent PK and PD across injection sites and ethnicities.

**Abbreviations**
AEs: adverse events
ALT: alanine transaminase
AST: aspartate aminotransferase
AUC: area under the plasma‐concentration time curve
BMI: body mass index
GCP: Good Clinical Practice
ICH: International Conference on Harmonization
IgG2: immunoglobulin G2
IV: intravenous
mAb: monoclonal antibody
PD: pharmacodynamics
PK: pharmacokinetics
SAEs: serious adverse events
SC: subcutaneously
sIL‐33: soluble interleukin 33
TMDD: target mediated drug disposition

## 1 INTRODUCTION

Severe asthma affects approximately 5%–10% of patients with asthma and is associated with frequent asthma exacerbations, decreased health‐related quality of life, and substantial symptom burden.^1^, ^2^, ^3^, ^4^, ^5^ Patients with severe asthma are at a greater risk of hospitalization and carry a higher socioeconomic burden compared with patients with mild or moderate asthma; treatment for this subgroup of patients represents a high proportion of total asthma‐related healthcare costs.^2^, ^3^, ^4^, ^5^ Asthma is a heterogeneous disease, with subtypes defined by their underlying pathophysiology and cytokine expression.^4^, ^6^, ^7^ In T‐helper cell type 2 (T2)‐driven asthma, inhaled pollutants, microbes, and allergens associate with the airway epithelium, leading to activation of mediators such as the alarmin interleukin (IL) 33.^6^ This leads to upregulation of IL‐4, IL‐5, and IL‐13, which can lead to activation of eosinophils. In non–T2‐driven asthma, eosinophil levels are not elevated, and the pathophysiology is poorly understood.^6^, ^8^ Most current biologic agents that are approved for the management of patients with severe asthma have demonstrated efficacy for T2‐driven disease; however, therapies targeting non–T2‐driven asthma are very limited.^6^, ^7^ Of note, tezepelumab, a monoclonal human antibody against thymic stromal lymphopoietin, shown to mediate both T2 and non–T2‐driven asthma, has recently been approved by the US Food and Drug Administration (FDA).^9^, ^10^ Inhibition of IL‐33 signaling via blockade of its receptor (IL‐33R), represents a potential novel treatment for severe asthma.^8^, ^9^ Agents targeting this mechanism could be expected to have effects on both types of disease because multiple cell types including mast cells, basophils, type 2 T‐helper cells, invariant natural killer cells and natural killer cells are involved in both T2‐driven and non–T2‐driven asthma and express IL‐33R.^8^

GSK3772847 (formerly CNTO 7160) is a human monoclonal immunoglobulin G2 (IgG2) sigma antibody that binds domain 1 of the cell surface IL‐33R. Three clinical trials have been conducted previously with GSK3772847 using intravenous (IV) administration (NCT02345928, NCT03207243, NCT03393806)^11^; however, the subcutaneous (SC) route is usually preferred by patients and can be more cost‐effective.^12^ The aim of this Phase 1 study (GSK ID 209635/NCT04366349) was to evaluate the safety, tolerability, pharmacokinetics, and pharmacodynamics of single ascending doses of GSK3772847, compared with placebo. GSK3772847 was administered SC in three different injection sites in healthy participants. Cohorts of Japanese and Chinese participants received GSK3772847 SC in the upper arm only.

## 2 METHODS

### 2.1 Study design and treatments

**FIGURE 1:**
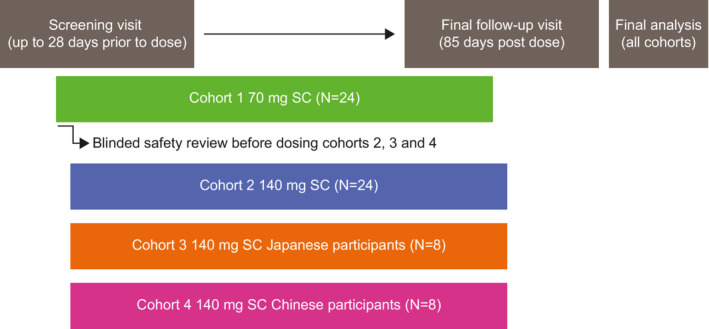
Study schema. SC, subcutaneous

This Phase 1, single‐center, randomized, placebo‐controlled, double‐blind, single ascending dose study in healthy participants was conducted in the United States from 21 July 2020 to 21 December 2020. Following a screening period (of up to 28 days), participants who met the eligibility criteria were assigned to one of four cohorts and were randomly allocated within each cohort to receive a single dose of either GSK3772847 or placebo SC (Figure 1). The site of injection was randomized to the upper arm, abdomen, or thigh for cohorts 1 and 2; with cohorts 3 and 4 receiving injections in the upper arm only. Before dosing in cohorts 2, 3, and 4, blinded safety data (including injection site data from dosing to a minimum of 48 h post‐dose) were reviewed from at least 20 participants who had received a dose in cohort 1.

On Day 1, participants were assigned a unique randomization number, encoding the participant's assignment to one of the four study arms according to the schedule generated prior to the study. In cohorts 1 and 2, 18 participants were randomly allocated to receive GSK3772847 70 mg (cohort 1) or GSK3772847 140 mg (cohort 2) SC, and six participants to receive placebo SC in each cohort. Participants in cohorts 1 and 2 were also randomized to one of three injection sites (upper arm, abdomen, or thigh). In cohort 3, six Japanese participants were randomly allocated to receive GSK3772847 140 mg SC, and two to receive placebo SC in the upper arm injection site. Finally, in cohort 4, six Chinese participants were randomized to receive GSK3772847 140 mg SC, and two to receive placebo SC in the upper arm injection site. Participants were admitted to the unit the day prior to dosing (Day 0) and were discharged after completion of assessments on Day 6. Participants attended follow‐up visits on Days 9, 15, 29, 43, 57, 71, and 85.

The study protocol was reviewed and approved by an institutional review board in accordance with the International Conference on Harmonisation (ICH), Good Clinical Practice (GCP) and relevant country‐specific requirements, all applicable patient privacy requirements, and the guiding principles of the current version of the Declaration of Helsinki. Investigators were trained to conduct the study in accordance with GCP and the study protocol and all patients provided written informed consent prior to study participation. All study staff involved in clinical assessments, including the investigator, sub‐investigators and other site staff, and the participants were blinded to the individual treatments allocated.

### 2.2 Participants

Eligible participants were healthy males and females aged between 18 and 50 years, with a body weight of 35–150 kg and a body mass index (BMI) of 18–32 kg/m^2^. Cohort 3 included Japanese participants only, and cohort 4 included Chinese participants only. Participants were excluded if their alanine transaminase (ALT) level was >2× the upper limit of normal or if their bilirubin was >1.5× the upper limit of normal. Participants were also excluded if they had current or chronic history of liver disease, ongoing or recurrent infections, a history of smoking within 6 months of study screening, or clinically significant electrocardiogram ECG findings.

The safety population included all randomized participants who received at least one dose of study intervention (analyzed based on treatment received). The PK population included all randomized participants who received at least one dose of study treatment, and for whom at least one post‐randomization PK sample was obtained, analyzed, and was measurable. The PD population included all randomized participants who received at least one dose of study treatment, and for whom at least one PD sample was obtained, analyzed, and was measurable.

### 2.3 Study endpoints and assessments

The primary safety endpoints were the incidence and frequency of adverse events (AEs) and serious adverse events (SAEs), including injection site reactions, and these were summarized descriptively by treatment group and cohort. Further primary endpoints were PK parameters including, but not limited to, area under the plasma‐concentration time curve (AUC), maximum plasma concentration (*C*_max_), time to *C*_max_ (*t*_max_) and terminal half‐life (*t*1/2) of GSK3772847, summarized by cohort and by injection site.

Secondary endpoints were the maximal decrease from baseline (the last assessment prior to the first study dose) in free soluble IL‐33 (sIL‐33); a maximal increase from baseline in total sIL‐33 levels in serum summarized by cohort (and injection site for cohorts 1 and 2); and the incidence and prevalence of anti‐GSK3772847 antibodies and plasma 4β‐hydroxycholesterol/cholesterol (4βOHC/C) ratio (pre‐treatment and following dosing of GSK3772847), summarized by cohort (and injection site for cohorts 1 and 2) over time.

Other endpoints included 12‐lead ECGs, clinical laboratory safety tests, vital signs, blood eosinophil levels, free and total sIL‐33 levels in serum, and GSK3772847 levels in the serum, summarized by cohort (and injection site for cohorts 1 and 2) over time. All endpoints were summarized descriptively.

### 2.4 Target engagement assessments

GSK3772847 binds domain 1 of transmembrane (IL‐33R) and soluble (sIL‐33R) forms of IL‐33R. Free sIL‐33 and total sIL‐33 concentration in serum were assessed using validated, specific, and sensitive electrochemiluminescent assays (Meso Scale Discovery).

### 2.5 Sample size and statistical analyses

The sample size was selected to allow estimation of the key PK parameters (AUC and *C*_max_) with adequate precision (1 standard error [SE], expected to be ~7% of the geometric mean for each dose [*n* = 18] assuming a coefficient of variation [CV] no greater than 30%) based on comparable monoclonal antibody (mAb) studies. This sample size was also deemed adequate to enable an understanding of the tolerability of GSK3772847 when dosed SC.

A total of 64 participants were planned to be randomized to receive GSK3772847 or placebo for a total of 18 evaluable participants on GSK3772847 in cohorts 1 and 2, six Japanese participants in cohort 3, and six Chinese participants in cohort 4. While this study was not designed to determine bioequivalence, the sample size for the critical evaluations of dose and injection site comparisons were in line with appropriate guidance, where the minimum number of participants should not be smaller than 12, (when combined across both doses).^13^ All endpoints were summarized descriptively.

### 2.6 Nomenclature of targets and ligands

Key protein targets and ligands in this article are hyperlinked to corresponding entries in http://www.guidetopharmacology.org, the common portal for data from the IUPHAR/BPS Guide to PHARMACOLOGY,^14^ and are permanently archived in the Concise Guide to PHARMACOLOGY 2019/20.^15^, ^16^, ^17^

## 3 RESULTS

### 3.1 Participant population

Patient disposition is shown in Table S1. A total of 90 participants were screened. Overall, 65 entered the study and 64 participants completed the study as planned. The demographic and baseline characteristics of study participants are presented in Table 1. Japanese participants (cohort 3) had the lowest mean (SD) BMI, 20.12 kg/m^2^ (1.493), and mean (SD) body weight, 49.50 kg (4.308), compared with participants from other study cohorts. One participant from cohort 2 withdrew consent on Day 8 due to personal reasons. This participant was replaced with another participant, allocated to the same randomized treatment but with a new randomization number, as specified in the protocol. All 65 participants who entered the study received the study medication as per the study design according to randomization.

**TABLE 1:** Participant demographics

Demographics	GSK3772847 Cohort 1			GSK3772847 Cohort 2			Placebo SC (Cohorts 1 and 2) *N* = 13	GSK3772847 Cohort 3	GSK3772847 Cohort 4	Placebo SC (Cohorts 3 and 4) *N* = 4
	70 mg SC (Abdomen) *N* = 6	70 mg SC (Thigh) *N* = 6	70 mg SC (Upper Arm) *N* = 6	140 mg SC (Abdomen) *N* = 6	140 mg SC (Thigh) *N* = 6	140 mg SC (Upper Arm) *N* = 6		Japanese 140 mg SC (Upper Arm) *N* = 6	Chinese 140 mg SC (Upper Arm) *N* = 6	
Age, years^a^, mean (SD)	34.8 (9.81)	31.7 (8.52)	30.7 (13.49)	30.8 (9.99)	33.5 (11.36)	30.7 (9.44)	36.2 (6.64)	41.7 (7.92)	42.5 (6.25)	38.5 (11.00)
Sex, female, *n* (%)	3 (50)	2 (33)	3 (50)	2 (33)	3 (50)	4 (67)	6 (46)	5 (83)	1 (17)	4 (100)
BMI, kg/m^2^, mean (SD)	26.33 (3.199)	26.80 (3.754)	26.77 (4.015)	26.45 (4.636)	26.97 (3.319)	23.47 (4.057)	25.31 (3.142)	20.12 (1.493)	26.10 (2.343)	21.55 (1.926)
Height, cm, mean (SD)	166.5 (11.22)	170.0 (11.64)	167.5 (8.73)	171.0 (8.74)	165.8 (6.79)	167.8 (7.28)	173.6 (8.66)	156.8 (5.53)	166.3 (8.78)	159.8 (3.10)
Weight, kg, mean (SD)	73.78 (15.799)	77.62 (13.514)	74.60 (8.199)	77.68 (16.840)	74.52 (12.788)	66.02 (11.211)	76.62 (13.253)	49.50 (4.308)	72.70 (11.854)	54.93 (4.165)
Ethnicity, *n* (%)										
Hispanic or Latino	3 (50)	3 (50)	5 (83)	4 (67)	3 (50)	4 (67)	3 (23)	0	0	0
Not Hispanic or Latino	3 (50)	3 (50)	1 (17)	2 (33)	3 (50)	2 (33)	10 (77)	6 (100)	6 (100)	4 (100)
Race, *n* (%)										
Asian	0	0	0	0	1 (17)	1 (17)	0	6 (100)	6 (100)	4 (100)
Central/South Asian Heritage	0	0	0	0	0	1 (17)	0	0	0	0
Japanese Heritage/East Asian Heritage/South‐East Asian Heritage	0	0	0	0	1 (17)	0	0	6 (100)	6 (100)	4 (100)
Black or African American	2 (33)	2 (33)	1 (17)	2 (33)	0	0	4 (31)	0	0	0
Native Hawaiian or Other Pacific Islander	0	0	0	0	0	0	1 (8)	0	0	0
White	4 (67)	4 (67)	5 (83)	4 (67)	5 (83)	5 (83)	8 (62)	0	0	0

Abbreviations: BMI, body mass index; SC, subcutaneous; SD, standard deviation.

^a^ For participants who attended a screening visit, age was calculated at the screening visit date. Only birth year was entered. Day and Month were imputed as 30 June.

### 3.2 Primary endpoints

#### 3.2.1 Safety results

In total, on‐treatment AEs were reported in 13 participants and post‐treatment AEs were reported in eight participants during the study (Table S2). No trend was observed in AEs related to the site of injection; one post‐treatment AE of COVID‐19 was reported. In cohort 2 (140 mg SC GSK3772847), a single drug‐related AE of mild intensity ‘headache’ was reported in one participant. An on‐treatment adverse event of special interest of ‘syncope’ was reported in two participants during the study and considered unrelated to the study medication. Most of the reported AEs were of mild intensity, considered as not related to the study medication by the investigator and were resolved without treatment. No deaths or SAEs were reported during the study and no other significant AEs leading to withdrawal from the study were reported. There were no clinically significant changes or abnormalities observed in hematology, vital signs, or ECG parameters during the study.

There were six reported clinical chemistry‐related AEs (ALT increased >33 IU/L and aspartate aminotransferase [AST] increased >32 IU/L) either pre‐dose or during the study. All events were reported as mild intensity, and the events were considered as not related to the study medication by the investigator.

There was a serious quality issue identified during the study, which was reported for investigation to the institutional review board. The investigation showed no impact of any clinical significance for any participants; further details can be found in the Supporting Information.

#### 3.2.2 Pharmacokinetic results

**FIGURE 2:**
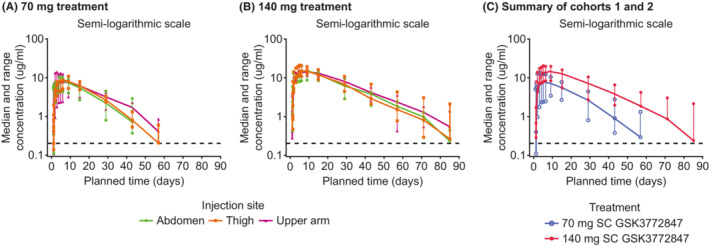
Median serum concentrations of GSK3772847 over time by dose and injection site (A, B) (PK population) and summary of median serum concentrations of GSK3772847 over time for cohorts 1 and 2 (PK population) (C). Dashed black line represents the lower limit of quantification (0.2 μg/ml). Error bars represent minimum and maximum values. PK, pharmacokinetic; SC, subcutaneous

Visual inspection of the median GSK3772847 serum concentration‐time profiles for both dose levels indicated no appreciable difference across injection sites (Figure 2A,B and Table S3). PK parameters per thigh, abdomen and upper arm injection sites were comparable with overlapping confidence intervals (Figure 2A,B and Table S3). Therefore, PK parameters were summarized across all injection sites by dose (Figure 2C and Table 2). Systemic exposure between cohort 1 (70 mg SC) and cohort 2 (140 mg SC), as measured by geometric mean AUC (0–*t*) increased in a slightly greater than dose‐proportional manner, with a 2.5‐fold increase in geometric mean for a twofold increase in dose (geometric mean: 4140 h*μg/ml [95% CI 3510–4880] vs. 10 300 [9080–17.0]) and geometric mean apparent terminal half‐life (*t*1/2) estimates of 243 h [95% CI 224–265] and 293 h [95% CI 256–335] for cohorts 1 and 2, respectively (Table 2 and Table S3). No formal statistical assessment of dose‐proportionality was performed, and we note that if corrected for dose there would be a slight overlap in the 95% CI for AUC. This observation is consistent with the significant non‐linearity in AUC, likely driven by target mediated drug disposition (TMDD), observed over the wider dose range studied with previous IV administration data.^11^ Consistent with this, the maximum plasma concentrations (*C*_max_) of GSK3772847 were approximately dose proportional (geometric mean: 7.8 μg/ml [95% CI 6.5–9.4] vs. 15.1 [13.4–17.0]; Table 2 and Table S3). Time of occurrence of *C*_max_ of GSK3772847 (*t*_max_) between cohorts 1 and 2 were comparable and attained 120 and 130 h post‐dose, respectively.

**TABLE 2:** Summary of derived GSK3772847 PK parameters for cohorts 1 and 2 (PK population)

PK parameters (units)	Cohort	*N*	*n*	Geometric mean	95% CI	%CVb
AUC (0–*t*) (h*μg/ml)	Cohort 1	18	18	4140	(3510, 4880)	34.1
	Cohort 2	18	18	10 300	(9080, 11 600)	25.2
*C*_max_ (μg/ml)	Cohort 1	18	18	7.8	(6.5, 9.4)	38.4
	Cohort 2	18	18	15.1	(13.4, 17.0)	24.5
*t*_max_ (h)^a^	Cohort 1	18	18	120	(48, 382)	
	Cohort 2	18	18	130	(72, 312)	
*t*½ (h)	Cohort 1	18	18	243	(224, 265)	17.1
	Cohort 2	18	18	293	(256, 335)	27.6

Abbreviations: %CVb, between subject variability; AUC, area under curve; CI, confidence interval; *C*_max_, maximum serum concentration; h, hours; *N*, number of participants; *n*, number of observations; PK, pharmacokinetics; *t*1/2, apparent terminal phase half‐life; *t*_max_, time of occurrence of *C*_max_.

^a^ *t*_max_ expressed as median and range.

**FIGURE 3:**
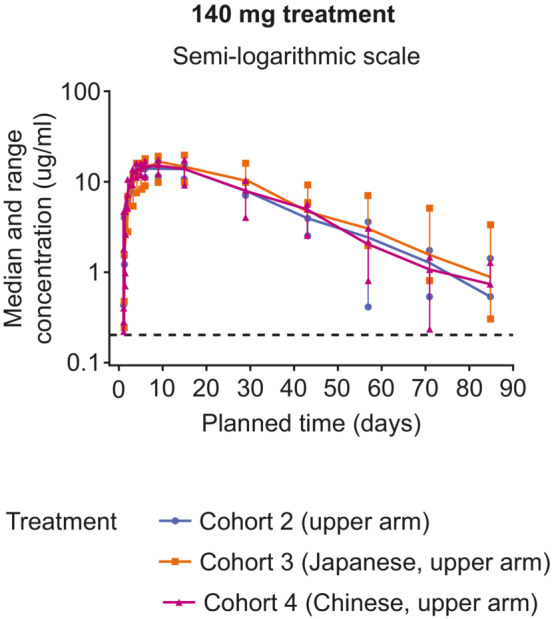
Median serum concentrations of GSK3772847 over time by cohort for 140 mg SC dose (PK population). Dashed black line represents the lower limit of quantification (0.2 μg/ml). Error bars represent minimum and maximum values. PK, pharmacokinetic; SC, subcutaneous

Overall, the geometric mean GSK3772847 serum concentration‐time profiles by cohort for 140 mg SC (upper arm) were comparable, with overlapping confidence intervals (Figure 3, Table 2, and Table S3). However, the median GSK3772847 serum concentration exposures were marginally but not significantly higher in cohort 3 (Japanese, upper arm) participants (geometric mean AUC (0–*t*) 13 400 h*μg/ml vs. 11 100 in cohort 2 and 11 300 in Chinese participants; Figure 3 and Table S3).

### 3.3 Secondary endpoints

#### 3.3.1 Pharmacodynamic results

**FIGURE 4:**
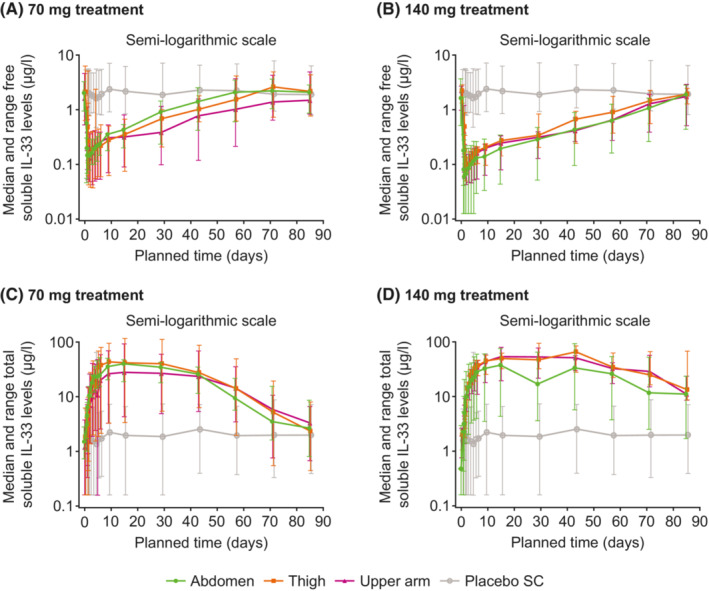
Median free soluble IL‐33 levels by dose, injection site, and placebo (a, b) and total soluble IL‐33 levels by dose, injections site, and placebo (c, d) (PD population). Error bars represent minimum and maximum values. PD, pharmacodynamic; SC subcutaneous

**FIGURE 5:**
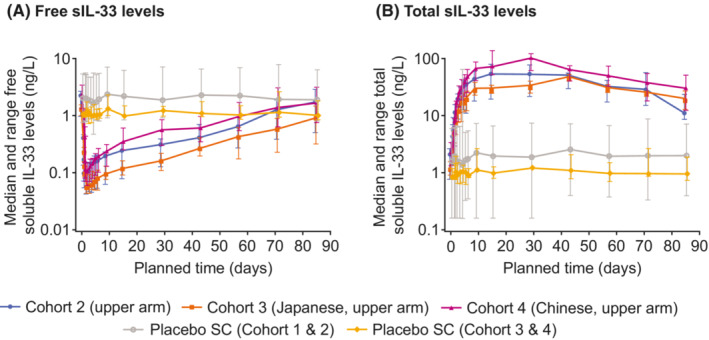
Median free and total soluble IL‐33 levels by cohort and placebo for 140 mg SC GSK3772847 dose (PD population). Error bars represent minimum and maximum values. IL‐33, interleukin 33; PD, pharmacodynamic; SC subcutaneous

A significant decrease in free sIL‐33 levels was seen in GSK3772847 cohorts as compared with placebo cohorts; this decrease was similar across ethnicities in cohorts 2, 3, and 4 over the study duration. The maximum decrease from baseline in free sIL‐33 levels (geometric mean) was >93% with GSK3772847 70 mg SC and >95% with GSK3772847 140 mg SC across all injection sites (Figure 4 and Table S4). The maximum increase from baseline in total sIL‐33 levels (geometric mean) was >2100% with GSK3772847 70 mg SC and >3200% with GSK3772847 140 mg SC across all injection sites (Table S5). A significant increase in total sIL‐33 levels was seen in GSK3772847 cohorts compared with placebo cohorts. The median total sIL‐33 level increased from baseline by the first post‐dose assessment (2 h post dose on Day 1) and appeared to pleateau from approximately Day 10 in all cohorts (Figures 4 and 5). The duration of this plateau was dose dependent (Figure 4) with time of maximal increase from Days 15 to 43 across cohorts/inhection sites. The increase in total sIL‐33 levels was similar across ethnicities in cohorts 2, 3, and 4 over the study duration (Figure 5).

#### 3.3.2 Immunogenicity

No participant receiving GSK3772847 in cohort 1, 2, or 3 tested positive for anti‐GSK3772847 binding antibodies in the confirmatory assay. One participant from cohort 4 (Chinese) confirmed positive results for anti‐GSK3772847 binding antibodies (titer 80) on Day 57 and Day 85.

#### 3.3.3 Plasma 4β‐hydroxycholesterol/cholesterol ratio

Plasma 4βOHC/C ratio to baseline was essentially unchanged (~1) over the study duration in all cohorts (Table S6).

## 4 DISCUSSION

This was the first study to administer GSK3772847 subcutaneously. GSK3772847 was administered in three injection sites (upper arm, abdomen, or thigh) in healthy volunteers, including cohorts of Chinese and Japanese participants. Both the 70 and 140 mg doses were generally well tolerated with no reported injection site reactions during the study. The majority of AEs were mild, considered unrelated to the study medication, resolved without treatment, and there was no trend related to the injection site. There were six clinical chemistry‐related AEs, which were also considered mild and unrelated to the study treatment. There were no SAEs or deaths.

Pharmacokinetics parameters were similar across the different injection sites with overlapping confidence intervals, indicating that the location of the injection site does not appear to affect drug availability. As expected, *C*_max_ and AUC (0–*t*) were higher at the 140 mg SC dose compared with the 70 mg SC dose. Whilst no formal statistical assessment of dose‐proportionality was performed, there does appear to be a reasonable approximation of dose proportionality for *C*_max_ and a slight non‐linearity for AUC, which are consistent with the expected TMDD. Consistent with this effect, target engagement was demonstrated with reduced free sIL‐33 concentrations and substantially increased total sIL‐33 concentrations compared with baseline.

*T*1/2 and *t*_max_ were comparable across 70 and 140 mg doses, with overlapping confidence intervals. PK parameters were also similar across ethnic groups with similar *t*_max_ and elimination profiles and overlapping confidence intervals. The median GSK3772847 serum concentration was slightly higher in cohort 3 (Japanese participants) than in cohorts 1, 2, and 4. Given the precedented impact of weight on mAb clearance, the lower mean weight in Cohort 3 is likely to have contributed to the small difference in GSK3772847 serum concentration. Overall, exposures were consistent with the expected 70%–80% for a mAb compared with previous IV administration data.^11^

In previous studies, PK and PD modeling suggested an IV administration of GSK3772847 1 mg/kg every 2 weeks would provide adequate systemic drug exposure for effective inhibition of the IL‐33R signaling pathway in 90% of participants.^11^ Assessments of serum concentrations over time suggests that the 70 mg SC dose may deliver a similar effect. Furthermore, the safety and tolerability profiles of GSK3772847 were similar in IV and SC administration routes.

As an endogenous marker for CYP3A4 activity, this study assessed changes in the plasma 4βOHC/C ratio. CYP3A4 is a member of the Cytochrome P450 family of enzymes, which catalyze reactions in the metabolism of a large proportion of drugs and contribute to variation in drug pharmacokinetics.^18^, ^19^, ^20^ 4βOHC is formed from CYP3A4 and plasma concentrations rise with increased CYP3A4 activity, making it an appropriate marker for CYP3A4 activity that can be assessed non‐invasively.^18^, ^19^ No changes were observed compared with baseline over the study duration.

Treatment with mAbs, including GSK3772847, may be associated with the development of antibodies against the therapeutic agent, and participants who develop antibodies to therapeutic proteins may be more likely to experience a hypersensitivity reaction or a decrease in efficacy. During this study, only one participant from cohort 4 had confirmed positive results for anti‐GSK3772847 binding antibodies on Day 57 and Day 85.

This was the first study to evaluate the SC dosing of GSK3772847 in healthy volunteers, including cohorts of Japanese and Chinese participants, which assessed the injection site, dose level, and ethnic group impact on PK and PD. Although the number of participants in this study was small, it was sufficiently powered to ensure an adequate understanding of the tolerability of GSK3772847 when dosed subcutaneously and would enable the estimation of the key PK parameters. In summary, GSK3772847 was well tolerated via the SC route, delivered an exposure and target engagement profile consistent with expectations based on data from the IV route^11^ and had consistent properties across injection sites and ethnicities.

## AUTHOR CONTRIBUTIONS

DF, SS, and CN were involved in the conception of the work, and EP, DF, SS, CQZ, and CN contributed to the analysis or interpretation of data. All authors drafted the work or revised it critically for important intellectual content, gave final approval of the version to be published, and agreed to be accountable for all aspects of the work.

## CONFLICT OF INTEREST

EP, SS, DF, CQZ, and CN are employees of GSK and hold stocks/shares.

## ETHICAL APPROVAL

The study protocol was reviewed and approved by an institutional review board in accordance with the International Conference on Harmonization Good Clinical Practice and relevant country‐specific requirements, all applicable patient privacy requirements, and the guiding principles of the current version of the Declaration of Helsinki.

## PATIENT CONSENT

All patients provided written informed consent prior to study participation.

## Supporting information


Data S1:

## Data Availability

Information on GSK's data sharing commitments and requesting access to anonymized individual participant data and associated documents can be found at https://protect‐eu.mimecast.com/s/ytNzCvo7ySy4kPwtQTepc?domain=clinicalstudydatarequest.com
