# Supplementary material for: Safety, tolerability, and pharmacokinetics of a single ascending subcutaneous dose of GSK3772847 in healthy participants
Source: Pharmacol Res Perspect. 2023 Feb 27;11(2):e01054. doi: 10.1002/prp2.1054 (PMC9969340; doi:10.1002/prp2.1054)
Supplement: Supplementary file 1 — Data S1: [file PRP2-11-e01054-s001.docx]

# Supplementary materials

Safety, tolerability and pharmacokinetics of a single ascending subcutaneous dose of GSK3772847 in healthy participants

Eleni Pefani PhD^1^, Sally Stone BSc (Hons)^2^, Chang-Qing Zhu PhD^2^, Carol Nunn MBBS^3^, David Fairman MSc^1^.

*^1^**GSK Medicines Research Centre, Stevenage, GSK, UK; ^2^GSK, Brentford, Middlesex, UK; ^3^GSK RD Respiratory R&D, Brentford, UK*

Supplementary Results: Protocol deviation

Supplementary Table 1: Participant disposition

Supplementary Table 2: Summary of AEs

Supplementary Table 3: Summary of derived GSK3772847 PK parameters by injection site and ethnicity (PK population)

Supplementary Table 4: Summary of maximal decrease from baseline in free soluble IL-33 concentration (µg/L) over the study duration by cohort and injection site (PD population)

Supplementary Table 5: Summary of maximal increase from baseline in total soluble IL-33 concentration (µg/L) over the study duration by cohort and injection site (PD population)

Supplementary Table 6: Summary of plasma 4βOH cholesterol/cholesterol ratio to baseline (PD population)

Supplementary Results

## Significant quality issue

A significant quality issue (SQI) was investigated by the medical monitor on 22-Jan-2021. During the study, white blood cell (WBC) values were blinded, whereas all other haematology values were unblinded to allow the investigator the ability to review the results. There was no documented evidence of the PI reviewing the haematology results post-screening because the blinded WBC values had not been separated from the other haematology values.

After identification of the issue, the clinical site’s unblinded staff redacted the WBC differential counts from each haematology report for all participants from Day 1 to Day 85. The review of redacted haematology report values for all participants was completed by the investigator on 26-Jan-2021. The review was documented in the site close out visit report. The investigator confirmed that there were no values of clinical significance for any participants.

Supplementary Table 1: Participant disposition

| Number of participants | Cohort 1*  GSK3772847 70 mg SC  N=18 | Cohort 2*  GSK3772847  140 mg SC  N=18 | Placebo SC, Cohorts 1 and 2* N=13 | Cohort 3 Japanese  GSK3772847  140 mg SC (Upper Arm)  N=6 | Cohort 4 Chinese  GSK3772847  140 mg SC (Upper Arm)  N=6 | Placebo SC, Cohorts 3 and 4 N=4 |
| --- | --- | --- | --- | --- | --- | --- |
| Number of participants planned, n† | 18 | 18 | 12 | 6 | 6 | 4 |
| Number of participants randomised, n | 18 | 18 | 13 | 6 | 6 | 4 |
| Number of participants included in safety population, n (%) | 18 (100) | 18 (100) | 13 (100) | 6 (100) | 6 (100) | 4 (100) |
| Number of participants included in PD population, n (%) | 18 (100) | 18 (100) | 13 (100) | 6 (100) | 6 (100) | 4 (100) |
| Number of participants included in PK population, n (%) | 18 (100) | 18 (100) | 0 | 6 (100) | 6 (100) | 0 |
| Number of participants completed as planned, n (%)‡ | 18 (100) | 18 (100) | 12 (92) | 6 (100) | 6 (100) | 4 (100) |
| Number of participants withdrawn (any reason), n (%) | 0 | 0 | 1 (8) | 0 | 0 | 0 |
| Primary reason for withdrawal, n (%) | | | | | | |
| Withdrew consent§ | 0 | 0 | 1 (8) | 0 | 0 | 0 |
| *For cohorts 1 and 2, the site of injection was randomised to the upper arm, thigh (N=6 for GSK3772847 and N=2 for Placebo), or abdomen (N=6 for GSK3772847, cohort 1: N=2 for placebo, cohort 2: N=3 for placebo). †Planned to complete the study. ‡Completed follow-up: Participant completed the 12-week follow-up period. §Particpant 100042 (from placebo cohort 2, injection site abdomen) was not able to continue the study due to personal reasons. This participant was replaced with another participant.  PD, pharmacodynamic; PK, pharmacokinetic; SC, subcutaneous | | | | | | |

Supplementary Table 2: Summary of AEs

|  | GSK3772847 Cohort 1 | | | | GSK3772847 Cohort 2 | | | |  | | GSK3772847 Cohort 3 | GSK3772847 Cohort 4 |  |
| --- | --- | --- | --- | --- | --- | --- | --- | --- | --- | --- | --- | --- | --- |
|  | 70 mg SC (Abdomen) N=6 | 70 mg SC (Thigh)  N=6 | 70 mg SC (Upper Arm)  N=6 | 140 mg SC (Abdomen) N=6 | | 140 mg SC (Thigh)  N=6 | 140 mg SC (Upper Arm)  N=6 | Placebo SC (Cohorts 1 and 2)  N=13 | | Japanese 140 mg SC (Upper Arm)  N=6 | | Chinese  140 mg SC (Upper Arm) N=6 | Placebo SC (Cohorts 3 and 4)  N=4 |
| On-treatment adverse events | | | | | | | | | | | | | |
| Any AE  Headache  Syncope  Arthropod bite  ALT increased  AST increased  Back pain  Vomiting | 0  0  0  0  0  0  0  0 | 1 (17)  1 (17)  0  0  0  0  1 (17)  0 | 1 (17)  0  0  1 (17)  0  0  1 (17)  0 | 2 (33)  2 (33)  1 (17)  0  0  0  0  1 (17) | | 1 (17)  0  1 (17)  0  0  0  0  0 | 0  0  0  0  0  0  0  0 | 3 (23)  2 (15)  0  1 (8)  0  0  0  0 | | 2 (33)  0  0  2 (33)  0  1 (17)  0  0 | | 2 (33)  0  0  0  2 (33)  0  0  0 | 1 (25)  0  0  1 (25)  0  0  0  0 |
| Post-treatment adverse events | | | | | | | | | | | | | |
| Any AE  ALT increased  AST increased  COVID-19  Pharyngitis streptococcal  Diarrhoea  Ligament sprain | 0  0  0  0  0  0  0 | 2 (33)  0  1 (17)  0  1 (17)  0  0 | 1 (17)  1 (17)  1 (17)  0  0  0  0 | 1 (17)  1 (17)  0  0  0  0  0 | | 1 (17)  0  0  0  0  0  1 (17) | 2 (33)  0  0  1 (17)  0  1 (17)  0 | 0  0  0  0  0  0  0 | | 0  0  0  0  0  0  0 | | 1 (17)  1 (17)  0  0  0  0  0 | 0  0  0  0  0  0  0 |
| Note. On-treatment adverse event definition: study treatment start date <= adverse event onset date <= study treatment stop date plus 28 days. Post-treatment adverse event definition: adverse event onset date > study treatment stop date plus 28 days  AE, adverse event; ALT, alanine aminotransferase; AST, aspartate aminotransferase; SC, subcutaneous | | | | | | | | | | | | | |

Supplementary Table 3: Summary of derived GSK3772847 PK parameters by injection site and ethnicity (PK population)

| PK parameters (units) | Cohort | N | n | Geometric mean | 95% CI | %CVb |
| --- | --- | --- | --- | --- | --- | --- |
| AUC(0–t) (h*µg/mL) | Cohort 1, Abdomen | 6 | 6 | 3770 | (2520, 5640) | 39.8 |
|  | Cohort 1, Thigh | 6 | 6 | 4430 | (3410, 5760) | 25.4 |
|  | Cohort 1, Upper Arm | 6 | 6 | 4250 | (2840, 6350) | 39.7 |
|  | Cohort 2, Abdomen | 6 | 6 | 9360 | (6590, 13300) | 34.4 |
|  | Cohort 2, Thigh | 6 | 6 | 10500 | (8060, 13600) | 25.3 |
|  | Cohort 2, Upper Arm | 6 | 6 | 11100 | (9570, 12800) | 13.9 |
|  | Cohort 3, Japanese participants, Upper Arm | 6 | 6 | 13400 | (9850, 18100) | 29.7 |
|  | Cohort 4, Chinese participants, Upper Arm | 6 | 6 | 11300 | (8680, 14700) | 25.4 |
| Cmax (ug/mL) | Cohort 1, Abdomen | 6 | 6 | 7.8 | (5.5, 11.0) | 33.4 |
|  | Cohort 1, Thigh | 6 | 6 | 8.1 | (6.4, 10.4) | 23.4 |
|  | Cohort 1, Upper Arm | 6 | 6 | 7.6 | (4.2, 13.5) | 59.7 |
|  | Cohort 2, Abdomen | 6 | 6 | 13.9 | (9.5, 20.4) | 37.5 |
|  | Cohort 2, Thigh | 6 | 6 | 16.4 | (13.4, 20.1) | 19.5 |
|  | Cohort 2, Upper Arm | 6 | 6 | 14.9 | (13.2, 16.8) | 11.3 |
|  | Cohort 3, Japanese participants, Upper Arm | 6 | 6 | 15.9 | (12.2, 20.7) | 25.5 |
|  | Cohort 4, Chinese participants, Upper Arm | 6 | 6 | 15.8 | (13.5, 18.5) | 15.2 |
| Tmax (h)† | Cohort 1, Abdomen | 6 | 6 | 120 | (72.0, 191) | - |
|  | Cohort 1, Thigh | 6 | 6 | 179 | (96.0, 313) | - |
|  | Cohort 1, Upper Arm | 6 | 6 | 144 | (48.0, 382) | - |
|  | Cohort 2, Abdomen | 6 | 6 | 130 | (72.0, 168) | - |
|  | Cohort 2, Thigh | 6 | 6 | 120 | (96.0, 192) | - |
|  | Cohort 2, Upper Arm | 6 | 6 | 169 | (72.1, 312) | - |
|  | Cohort 3, Japanese participants, Upper Arm | 6 | 6 | 146 | (145, 313) | - |
|  | Cohort 4, Chinese participants, Upper Arm | 6 | 6 | 205 | (72.0, 382) | - |
| t½ (h) | Cohort 1, Abdomen | 6 | 6 | 247 | (204, 299) | 18.4 |
|  | Cohort 1, Thigh | 6 | 6 | 236 | (213, 261) | 9.6 |
|  | Cohort 1, Upper Arm | 6 | 6 | 247 | (194, 315) | 23.4 |
|  | Cohort 2, Abdomen | 6 | 6 | 281 | (209, 378) | 28.8 |
|  | Cohort 2, Thigh | 6 | 6 | 283 | (202, 396) | 33.0 |
|  | Cohort 2, Upper Arm | 6 | 6 | 317 | (248, 406) | 23.7 |
|  | Cohort 3, Japanese participants, Upper Arm | 6 | 6 | 343 | (236, 498) | 36.8 |
|  | Cohort 4, Chinese participants, Upper Arm | 6 | 6 | 292 | (232, 367) | 22.2 |
| †tmax expressed as median and range  %CVb, between subject variability; AUC, area under curve; Cmax, maximum serum concentration; N, number of participants; n, number of observations; PK, pharmacokinetics; t1/2, apparent terminal phase half-life; tmax, time of occurrence of Cmax | | | | | | |

Supplementary Table 4: Summary of maximal decrease from baseline in free soluble IL-33 concentration (µg/L) over the study duration by cohort and injection site (PD population)

|  | GSK3772847 Cohort 1 | | | GSK3772847 Cohort 2 | | |  | GSK3772847 Cohort 3 | GSK3772847 Cohort 4 |  |
| --- | --- | --- | --- | --- | --- | --- | --- | --- | --- | --- |
|  | 70 mg SC (Abdomen) N=6 | 70 mg SC (Thigh)  N=6 | 70 mg SC (Upper Arm)  N=6 | 140 mg SC (Abdomen) N=6 | 140 mg SC (Thigh)  N=6 | 140 mg SC (Upper Arm)  N=6 | Placebo SC (Cohorts 1 and 2)  N=13 | Japanese 140 mg SC (Upper Arm)  N=6 | Chinese 140 mg SC (Upper Arm) N=6 | Placebo SC (Cohorts 3 and 4)  N=4 |
| Maximal decrease – ratio to baseline | | | | | | | | | | |
| n* | 6 | 6 | 6 | 6 | 6 | 6 | 11 | 6 | 6 | 4 |
| Geometric mean | 0.0541 | 0.0699 | 0.0632 | 0.0374 | 0.0430 | 0.0450 | 0.7573 | 0.0453 | 0.0433 | 0.8073 |
| 95% CI | (0.0320, 0.0884) | (0.0553, 0.0872) | (0.0155, 0.1363) | (0.0254, 0.0531) | (0.0278, 0.0660) | (0.0348, 0.0577) | (0.6586, 0.9660) | (0.0311, 0.0638) | (0.0399, 0.0469) | (0.7150, 0.9028) |
| Maximal decrease – % change from baseline | | | | | | | | | | |
| n* | 6 | 6 | 6 | 6 | 6 | 6 | 11 | 6 | 6 | 4 |
| Geometric mean | 94.59 | 93.01 | 93.68 | 96.26 | 95.70 | 95.50 | 24.27 | 95.47 | 95.67 | 19.27 |
| 95% CI | (96.80, 91.16) | (94.47, 91.28) | (98.45, 86.37) | (97.46, 94.69) | (97.22, 93.40) | (96.52, 94.23) | (34.14, 3.40) | (96.89, 93.62) | (96.01, 95.31) | (28.50, 9.72) |
| *Number of participants with analysable data. Baseline is the most recent recorded value before dosing on Day 1. % Change is calculated as (Ratio to baseline-1)×100. %CV = sqrt(exp(SD of log values^2)-1)×100.Values below the lower level of quantification (<0.025 µg/L) are imputed as LLOQ×0.5. Participant 100042 (from Placebo cohort 2, injection site abdomen) was not able to continue the study due to personal reasons; this participant was replaced with another participant.  CI, confidence interval; PD, pharmacodynamic; SC, subcutaneous | | | | | | | | | | |

Supplementary Table 5: Summary of maximal increase from baseline in total soluble IL-33 concentration (µg/L) over the study duration by cohort and injection site (PD population)

|  | GSK3772847 Cohort 1 | | | | GSK3772847 Cohort 2 | | |  | GSK3772847 Cohort 3 | GSK3772847 Cohort 4 |  |
| --- | --- | --- | --- | --- | --- | --- | --- | --- | --- | --- | --- |
|  | 70 mg SC (Abdomen) N=6 | 70 mg SC (Thigh)  N=6 | 70 mg SC (Upper Arm)  N=6 | 140 mg SC (Abdomen) N=6 | | 140 mg SC (Thigh)  N=6 | 140 mg SC (Upper Arm)  N=6 | Placebo SC (Cohorts 1 and 2)  N=13 | Japanese 140 mg SC (Upper Arm)  N=6 | Chinese  140 mg SC (Upper Arm) N=6 | Placebo SC (Cohorts 3 and 4)  N=4 |
| Maximal increase – ratio to baseline | | | | | | | | | | | |
| n* | 6 | 6 | 6 | 6 | | 6 | 6 | 12 | 6 | 6 | 4 |
| Geometric mean | 24.5670 | 29.4166 | 22.8293 | 54.4538 | | 37.1934 | 33.2365 | 2.0573 | 39.1039 | 45.7791 | 1.7203 |
| 95% CI | (18.4640, 31.9812) | (17.9466, 45.1892) | (16.8099, 30.3022) | (-77.2597, 296.6989) | | (28.0104, 48.3664) | (23.1089, 46.1660) | (-1.8417, 10.8808) | (29.7883, 50.6161) | (35.8528, 57.6231) | (1.1951, 2.2921) |
| Maximal increase – % change from baseline | | | | | | | | | | | |
| n* | 6 | 6 | 6 | 6 | | 6 | 6 | 12 | 6 | 6 | 4 |
| Geometric mean | 2357.60 | 2841.66 | 2182.93 | 5345.38 | | 3619.34 | 3223.65 | 105.73 | 3810.39 | 4477.91 | 72.03 |
| 95% CI | (1746.40, 3098.12) | (1694.66, 4418.92) | (1580.99, 2930.22) | (-7825.97, 29569.89) | | (2701.04, 4736.64) | (2210.89, 4516.60) | (-284.17, 988.08) | (2878.83, 4961.61) | (3485.28, 5662.31) | (19.51, 129.21) |
| *Number of participants with analysable data. Baseline is the most recent recorded value before dosing on Day 1. % Change is calculated as (Ratio to baseline-1)×100. %CV = sqrt(exp(SD of log values^2)-1)100. Values below the lower level of quantification (<0.32 µg/L) are imputed as LLOQ×0.5. Participant 100042 (from Placebo cohort 2, injection site abdomen) was not able to continue the study due to personal reasons; this participant was replaced with another participant.  CI, confidence interval; PD, pharmacodynamic; SC, subcutaneous | | | | | | | | | | | |

| Visit | Cohort 1 GSK3772847 70 mg SC (N=18) | | Cohort 2 GSK3772847  140 mg SC (N=18) | | Placebo SC (Cohorts 1 and 2) (N=13) | | Cohort 3 Japanese GSK3772847 140 mg SC (Upper arm) (N=6) | | Cohort 4 Chinese GSK3772847 140 mg SC (Upper arm) (N=6) | | Placebo SC (Cohorts 3 and 4) (N=4) | |
| --- | --- | --- | --- | --- | --- | --- | --- | --- | --- | --- | --- | --- |
|  | n | Geometric mean (95% CI) | n | Geometric mean (95% CI) | n | Geometric mean (95% CI) | n | Geometric mean (95% CI) | n | Geometric mean (95% CI) | n | Geometric mean (95% CI) |
| Day 5 | 18 | 0.998 (0.945, 1.053) | 18 | 1.092 (0.976, 1.221) | 13 | 1.117 (0.979, 1.274) | 6 | 1.051 (0.951, 1.163) | 6 | 1.192 (0.941, 1.511) | 4 | 1.062 (0.580, 1.944) |
| Day 15 | 18 | 0.992 (0.914, 1.076) | 18 | 1.092 (0.958, 1.244) | 11 | 1.021 (0.833, 1.180) | 6 | 1.054 (0.892, 1.244) | 6 | 1.285 (1.050, 1.572) | 4 | 1.106 (0.790, 1.547) |
| Day 29 | 18 | 0.980 (0.912, 1.052) | 17 | 1.200 (1.033, 1.394) | 12 | 1.119 (0.942, 1.329) | 6 | 1.041 (0.893, 1.215) | 6 | 1.488 (1.162, 1.906) | 4 | 1.143 (0.854, 1.528) |
| Day 85/ Early Withdrawal | 18 | 1.032 (0.941, 1.132) | 18 | 0.901 (0.798, 1.017) | 12 | 0.923 (0.821, 1.037) | 6 | 0.826 (0.727, 0.939) | 6 | 0.946 (0.723, 1.237) | 4 | 0.930 (0.701, 1.235) |
| Note. The baseline value is the latest pre-dose assessment. Baseline geometric mean for Day X is the geometric mean of the baseline values of all participants with a non-missing value at Day X. Three participants had their blood samples collected in a fasted state at the following visits: Participant 100015 at Day 15; Participant 100024 at Day 85; Participant 100029 at Day 29.  4βOH; 4β-Hydroxy; CI, confidence interval; PD, pharmacodynamic; SC, subcutaneous | | | | | | | | | | | | |

Supplementary Table 6: Summary of plasma 4βOH cholesterol/cholesterol ratio to baseline (PD population)
